# Phosphorus source driving the soil microbial interactions and improving sugarcane development

**DOI:** 10.1038/s41598-019-40910-1

**Published:** 2019-03-13

**Authors:** Thiago Gumiere, Alain N. Rousseau, Diogo Paes da Costa, Alice Cassetari, Simone Raposo Cotta, Fernando Dini Andreote, Silvio J. Gumiere, Paulo Sergio Pavinato

**Affiliations:** 1 Department of Soil Science, Luiz de Queiroz College of Agriculture, University of São Paulo, ESALQ/USP, Av. Pádua Dias, 11, CP 09, 13418-900 Piracicaba, São Paulo Brazil; 2 Institut National de la Recherche Scientifique, Centre Eau Terre Environnement. 490, rue de la Couronne, Quebec City, QC G1K 9A9 Canada; 3 Department of Soil and Agri-Food Engineering, Laval University, 2325 Rue de l’Université, Quebec City, QC G1V 0A6 Canada

## Abstract

The world demand for phosphate has gradually increased over the last decades, currently achieving alarming levels considering available rock reserves. The use of soil microorganisms, such as arbuscular mycorrhizal fungi (AMF), has been suggested as a promising alternative to improve phosphorus-use efficiency. However, the effect of the source of phosphorus on the interactions within the soil microbial community remains unclear. Here, we evaluated the links between the total dry matter content of sugarcane and the interactions within the soil microbial community under different phosphate sources, with/without AMF inoculation. The phosphate sources were Simple Superphosphate (SS, 18% of P_2_O_5_), Catalão rock phosphate (CA, 2.93% of P_2_O_5_) and Bayovar rock phosphate (BA, 14% of P_2_O_5_). The results indicated that the BA source led to the largest total dry matter content. The phosphate source affected total dry matter and the structure of the soil microbial communities. The bacterial interactions increased across sources with high percentage of P_2_O_5_, while the fungal interactions decreased. The interactions between bacterial and fungal microorganisms allowed to identify the percentage of P_2_O_5_ resulting in the highest total sugarcane dry matter. Our findings suggested the soil microbial interactions as a potential microbial indicator helping to improve the agricultural management.

## Introduction

The growth and the production of agricultural crops are mostly related and limited by the presence and available forms of nutrients in the soil^1^. Mineral fertilizers have been vastly used across different crops around the world, reducing the currently known rock reserves to alarming levels^2^. The world demand for nutrients such as nitrogen (N), potash (K_2_O) and phosphate (P_2_O_5_) was 185 million tons in 2015, and it is estimated to reach 202 million tons by 2020^3^. The demand for phosphate, for example, has reached 2 million tons over the last three years^3^. Given this situation, there is a need for a more efficient use of nutrients such as nitrogen^4^, potassium^5^ and, especially, phosphorus^6^ while reducing the environmental impacts^7^.

For plants in general, including sugarcane, one of the most important crops in Brazil, phosphorus is a key element in cell processes such as energy, photosynthesis and sugar transformation^8^. However, most soils have naturally low total phosphorus content. Moreover, some soils have elements that form complexes reducing the availability of phosphorus to plants^9^. Thus, efforts have been made on alternatives to improve the efficiency of phosphorus fertilizers. Albuquerque *et al*.^10^ tested different sources and doses of phosphorus in sugarcane production, and observed that Bayóvar reactive rock phosphate led to increases in stem diameter and dry matter at 120 days. Meanwhile, it is believed that certain microorganisms may improve phosphorus efficiency in the soil-plant system.

Mycorrhizal fungi are described as a crucial microbial group in soil systems, presenting mutualistic association with plants^11^. The occurrence of this group is considered one of the most important microbial indicators of soil quality^12^. The symbiotic association, known as mycorrhiza, improves plant nutrient uptake^13^ and water absorption^14^, reduces the effects of environmental stress on plants^15^, and also protects plants against pathogens^16,17^. Streitwolf-engel *et al*.^18^ observed that arbuscular mycorrhizal fungi (AMF) can increase phosphorus uptake, and plant biodiversity. This suggests AMF may be a major contributor to plant productivity and variability. Surprisingly, the effects of different sources of phosphorus on the colonization of AMF taxa, or even on the soil microbial community, have not been closely examined for major crops, such as sugarcane.

The mycorrhiza association between fungal hypha and plant roots, can lead to the establishment of a high interactive zone defined as mycorrhizosphere^19^. As reviewed by Barea *et al*.^20^, the mycorrhizosphere can be viewed as an interaction region, presenting a high production of glycoprotein and other composts that may stimulate other soil microorganisms such as the plant growth promoting rhizobacteria (PGPR). The comprehension of soil microbial interactions, especially those between bacterial and mycorrhizal fungal communities, is considered essential to develop sustainable crop production systems^21,22^. Furthermore, Menezes *et al*.^23^ addressed the importance of soil microbial interaction studies, suggesting experiments with different soil managements to determine the role of fungal-bacterial interactive.

In this sense, network analysis has become an important tool to evaluate microbial interactions across environmental systems^24^. Spearman and Pearson correlations have been used in microbial community interaction studies^25^, suggesting the quality and stability of different environmental systems^26^. Using a *fingerprinting* method and Pearson correlation, Morriën *et al*.^27^ suggested that network interactions of soil biota are correlated with soil carbon cycling across natural areas under restoration. Thus, network analysis has the potential to be used as a biological indicator for environmental systems^12^ such as cropping systems^28^. However, there is a current paucity of well-controlled studies on the use of microbial interactions to improve fertilization efficiency in cropping systems.

Here, our objective was to evaluate the effect of three different sources of phosphorus on sugarcane total dry matter and soil microbial interactions, considering the presence or absence of AMF inoculation. The control treatments included simple superphosphate (SS, 18.0% of P_2_O_5_), Catalão rock phosphate - Brazil (CA, 2.93% of P_2_O_5_), Bayovar rock phosphate - Peru (BA, 14.0% of P_2_O_5_) sources, along with a control treatment (no phosphorus fertilizer addition). The greenhouse experiment was performed with the same sugarcane cultivar during 120 days. The experimental pots were fertilized with the equivalent quantity of 60 mg of P_2_O_5_ per kg of soil for each phosphate source. Our hypotheses were: (*i*) there exists a negative correlation between the colonization of AMF and the percentage of P_2_O_5_ associated with the source; (*ii*) the source with high percentage of P_2_O_5_ increases the soil microbial interactions; and (*iii*) the interactions between bacterial and fungal communities are positively correlated with the percentage of P_2_O_5_ of the source and the production of sugarcane dry matter.

## Results

### Dry matter, colonization index and leaf nutrient content

**Figure 1:**
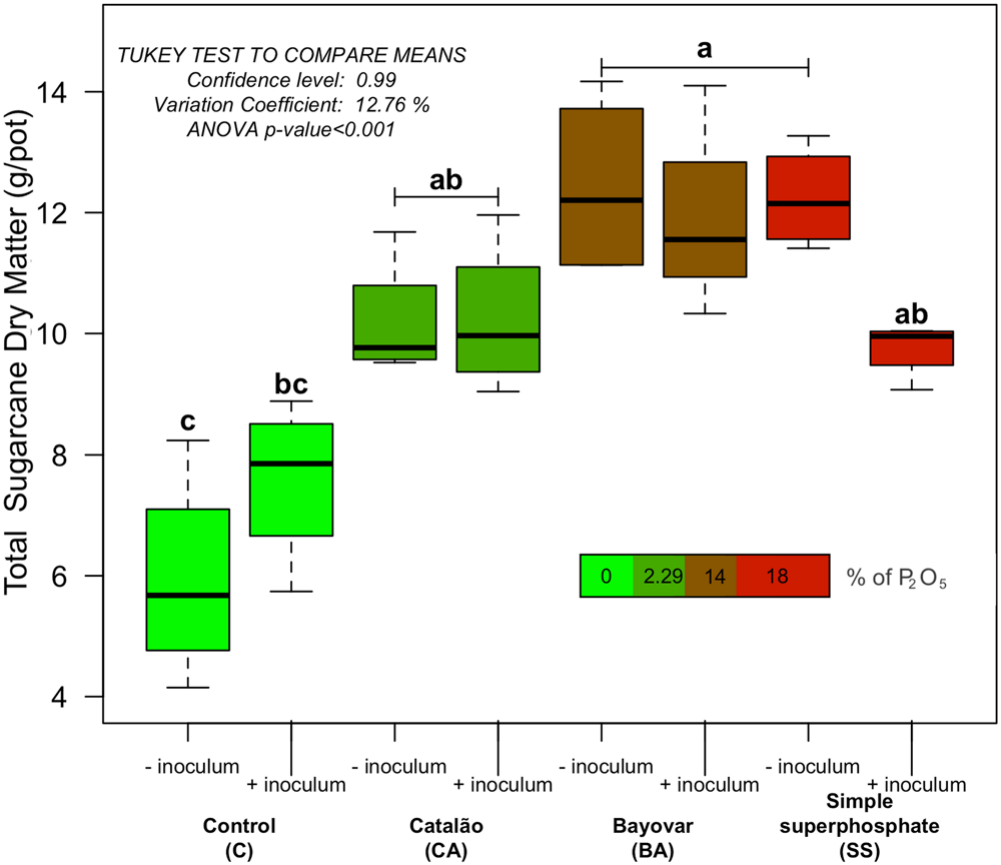
Boxplot of total dry matter (gram per pot) of sugarcane plants across phosphate sources in the presence or absence of mycorrhizal fungi inoculation. The bar colour indicates the percentage of P_2_O_5_ within each different phosphate source. The averages were compared by Tukey test (p-value < 0.001).

The total dry matter of sugarcane was substantially affected by the phosphate sources (Fig. 1). As expected, the increase in soluble phosphate led to larger values of total dry matter. We observed that the addition of AMF increased the total dry matter in the control, but decreased in the SS treatment. Large averages of total dry matter values were observed under BA with inoculum (11.9 g/vase), BA without inoculum (12.4 g/vase) and SS without inoculum (12.2 g/vase). Detail information about shoot and root individual dry matters can be found in Figs S1 and S2. The distinct phosphate sources showed a negative effect on the colonization index (Fig. S3), control with inoculum showing an average of 43% of roots colonized. The increase in percentage of P_2_O_5_ affected the root colonization index negatively, averaging 35% in CA, 23% in BA, and 16% in SS treatments. The decreased pattern was confirmed by linear regression (red line in Fig. S3) with R^2^ = 0.9924, and p-value < 0.001. Colonization was not observed in treatments without inoculum addition.

Nitrogen (N), phosphorus (P), and potassium (K) contents in plant tissue are presented in Table S2. Sugarcane leaves in control treatment showed the smallest value of P (0.69 g kg^−1^), with the largest value being observed in SS (1.3 g kg^−1^). For N, the control showed 10.71 g kg^−1^, which decreased across the treatments. Also, no significant variation in N content associated with CA, BA and SS treatments (ANOVA, p-value > 0.05) was observed. The K leaf content did not show a clear pattern, presenting largest value in CA (14.15 g kg^−1^) and lower values in BA and Control (12.43 g kg^−1^). The N, P, and K contents in plant tissue were higher in the treatments with AMF inoculation than in treatments without AMF inoculation (ANOVA, p-value < 0.05).

### Structures and microbial interactions of bacterial and fungal communities

**Figure 2:**
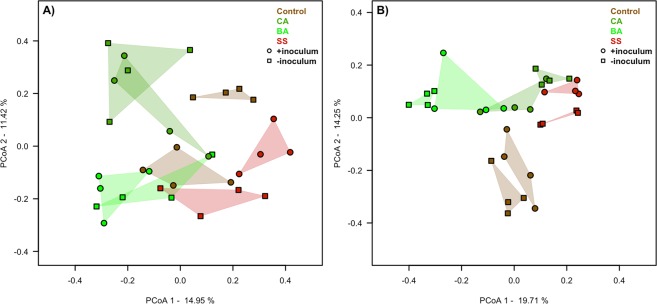
Principal coordinate analysis of bacterial (**A**) and fungal (**B**) communities. The colours indicate the phosphate sources in the presence (⚬) or absence (▫) of mycorrhizal fungi inoculation.

**Figure 3:**
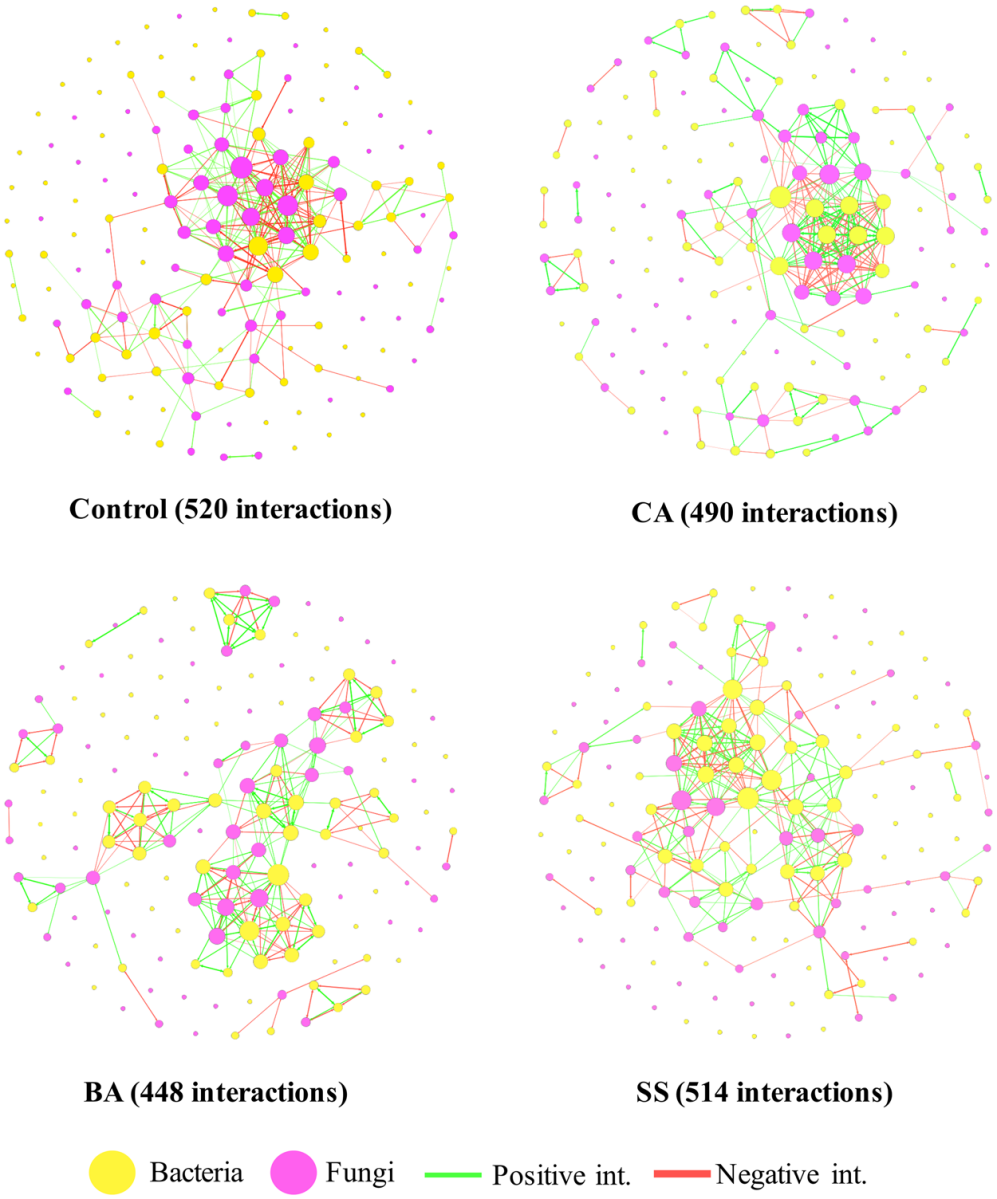
The network of bacterial (yellow circle) and fungal (purple circle) interaction obtained from Pearson and Spearman correlations (p-value < 0.05) for each phosphate source. Green lines depict positive correlations while those red illustrate negative correlations. The total number of microbial interactions is indicated for each phosphate treatment (Control, CA, BA, and SS).

The structure of bacterial and fungal communities showed a high correlation with phosphate sources and also with the AMF colonization (Fig. 2A,B). The DGGE gels (Fig. S4), and cluster analyses (Figs S5 and S6) of the bacterial and fungal communities are included in the supplementary material. The PERMANOVA analyses (Table S3) indicated that the phosphate sources explained 39.1% of the bacteria and 45.77% of the fungal variability. The addition of AMF inoculum had a correlation with soil bacteria community (41%), but it was not significantly correlated with the soil fungal community (PERMANOVA, p-value > 0.05). The results of soil microbial interactions indicated an interesting variability across phosphate sources (Fig. 3) and inoculum addition (Fig. S7). In Fig. 3, we observe that the total number of connections (edges of Spearman and Pearson correlations) followed Control > SS > CA > BA. Table 1 indicates that the BA treatment had the smallest number of interactions (edges = 448, and density = 0.018), but the largest Positive/Negative ratio (1.33), and modularity (0.707). Besides the high microbial connections (edges), the other phosphate sources had smaller Positive/Negative ratios than that associated with the BA treatment, especially the CA treatment which had more negative than positive interactions (ratio = 0.87).

**Table 1:** Topological parameters of network analysis based on Spearman and Pearson correlations.

	Treatments					
	Control	CA	BA	SS	+Inoculum	−Inoculum
Total Nodes	157	157	157	157	157	157
*Interacting Nodes*	86	100	80	86	110	102
*Bacteria Nodes*	48.8%	55.0%	57.5%	58.1%	57.27%	59.80%
*Fungi Nodes*	51.2%	45.0%	42.5%	41.9%	42.73%	40.20%
Total Edges	520	490	448	514	1024	924
*Positives*	278	228	256	282	536	516
*Negatives*	242	262	192	232	488	408
*Positive/Negative ratio*	1.15	0.87	1.33	1.22	1.10	1.26
*Bacteria-Bacteria* ^*(a)*^	64	120	156	222	318	336
*Fungi-Fungi*	200	120	84	66	256	162
*Bacteria-Fungi*	256	250	208	226	450	426
Average Degree^*(b)*^	12.09	9.8	11.2	11.95	9.309	9.06
Graph Density^*(c)*^	0.021	0.049	0.018	0.021	0.085	0.09
Number of Communities^(d)^	11	19	12	12	6	6
Modularity^*(e)*^	0.436	0.562	0.707	0.541	0.463	0.395
Average Clustering Coefficient^*(f)*^	0.219	0.292	0.315	0.261	0.349	0.263
Avg. Path Length^*(g)*^	3.381	4.48	3.97	3.7	2.74	2.8
Diameter^*(h)*^	9	12	9	10	7	6

The treatments were control (no addition of phosphorus fertilizer), phosphate sources (CA – Catalão rock phosphate with 2.29% of P_2_O_5_; BA – Bayovar rock phosphate with 14% of P_2_O_5_; SS – simple superphosphate with 18% of P_2_O_5_), and addition (+inoculum) absence (−inoculum) of mycorrhizal fungi. The edges presented were filtered by Spearman and Pearson p-value < 0.05.

^(a)^Edges filtered by exclusive correlation between bacteria-bacteria. It was also evaluated for fungi-fungi and bacteria-fungi;

^(b)^Average number of connections the one node presents with other nodes in the network;

^(c)^Proportion between the presented number of connections (edges) and the potential connection, which could exist between two nodes;

^(d)^Number of connected communities based on the Spinglass Algorithm^59^;

^(e)^Indicates the level of connections within each community classified. Values higher than 0.4 indicates stronger connections between the groups^61^;

^(f)^Indicates the degree of nodes that tend to cluster together;

^(g)^Indicates the average of minimal distances between all pairs of nodes;

^(h)^Indicates the average of network distance between all pairs of nodes.

Comparing the number of connections between the bacteria and bacteria microorganisms (bacteria-bacteria), we observed an increase related to the percentage of P_2_O_5_. When comparing the Control to the different treatments, there is an increase in the number of fungi-fungi interactions. The exclusive connections between bacteria and fungi microorganisms (bacteria-fungi) had similar patterns for the total edges, the BA treatment having the smallest number of connections (edges = 208). Across the control and phosphate sources, we observed that the AMF inoculation increased the number of connections (edges and degree) and the modularity, but reduced the Positive/Negative ratio from 1.26 to 1.10.

### Regressions between microbial interactions, total dry matter and phosphorus source

**Figure 4:**
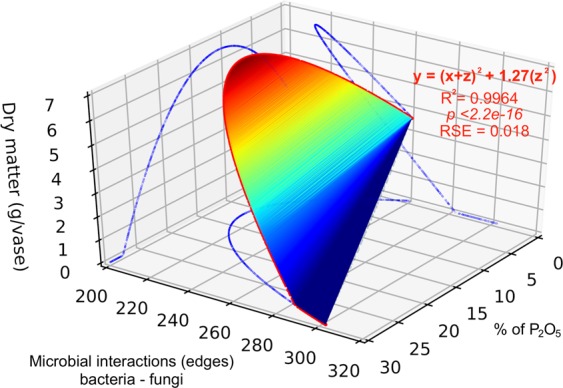
3D-plot of predicted correlation based on exponential regression curve between sugarcane total dry matter (gram per pot), percentage of P_2_O_5_ of each source, and number of bacteria-fungi interactions. The surface indicates the predicted values of total dry matter (gram per pot), from highest (red) to lowest values (blue). The red points indicate the exponential trendline (R^2^.adj = 0.9963, p-value < 2.2e^−16^). The blue shadows indicate 2D-correlations between the three factors.

The patterns observed for microbial interactions (edges) were correlated with the total dry matter of sugarcane and phosphate treatments by linear regression and exponential regression curves. We observed a significant correlation when fitting an exponential regression curve between total dry matter and soluble phosphate percentage (Fig. S8), with R^2^ = 0.67 and p-value < 0.001. A linear regression explained the correlation between bacteria-bacteria interactions and phosphorus source (R^2^ = 0.89, p < 0.001), while an exponential regression depicted well the fungi-fungi (R^2^ = 0.9, p < 0.001) and bacteria-fungi (R^2^ = 0.9, p < 0.001) interactions with the sources (Fig. S9). The linear and exponential regressions confirmed the increasing pattern observed for bacterial-bacterial interactions and the decreasing pattern for fungal-fungal interactions across the phosphates sources. Furthermore, the exponential curve of bacteria-fungi interactions showed the lowest relative standard error (RSE = 6.4), which was used as a parameter to select this exponential curve for the simulation analysis. The simulation analysis correlated the results obtained from the exponential regression curve of sugarcane total dry matter (Fig. S8) and bacteria-fungi interactions (Fig. S9) across phosphate sources. In Fig. 4, the surface indicates that the largest total dry matter (6.57 g per pot) is correlated with 217 edges of bacteria-fungi interactions and 11.09% of soluble P_2_O_5_. This exponential curve based on simulated results had R^2^ values of 0.9965 with p-value < 0.001.

## Discussion

Improving fertilizer-use efficiency has been considered as one of the most important challenges of the 21^st^ century^2,7^. The use of reactive rock phosphates has reduced fertilizer applications, inducing environmental benefits such as no wet acidification process and recovering nutrient plant efficiency^29^. Soltangheisi *et al*.^30^ observed that the use of rock phosphate increased the proportion of accumulated inorganic P in the soil, which was higher than the P proportion obtained by the use of SS fertilizer. Several reports have shown a positive effect of the AMF and the soil bacterial community on the rock phosphorus solubilization for different plants, such as maize^31^, wheat^32^, and alfafa^33^. However, the effect of different phosphorus sources on the AMF colonization and the soil microbial interactions had yet to be clearly illustrated. Thus in this study, we evaluated the effect of three phosphorus fertilizers on the AMF colonization and the soil microbial interactions, demonstrating their association with total dry matter.

The BA source improved the production of sugarcane dry matter in both treatments with/without AMF inoculation, confirming previously reported results for sugarcane^10^. However, an interesting decrease in the total dry matter under the SS source was observed with the addition of AMF inoculum. This negative effect of mycorrhizal colonization, which explicitly occurred with the SS source, could be related to the net carbon costs for fungal maintenance and growth, which exceeded the net benefits obtained of the host plant under high nutrient supply^34^. Otherwise, the reduction in total dry matter in the treatments with AMF inoculation was not detected under the other phosphate sources (CA and BA). These results not only confirm the negative effect of phosphate acidulated fertilizers on the AMF colonization^35^, such as SS, but also indicate an antagonism effect between source and inoculum resulting in a reduction in total dry mass.

The phosphate sources explained 39.1% and 45.77% of the bacteria and fungi variability, respectively. These results, obtained by a *fingerprinting* technique (DGGE), agreed well with the observations reported by Silva *et al*.^36^. Using next-generation sequencing, the authors observed alterations in soil microbial community composition with respect to the type of phosphate fertilization. Furthermore, the AMF inoculation showed a strong effect on the soil bacterial community, explaining 41% of their variability. This fact may be associated with the recruitment of bacteria by AMF, where the hypha-recruited bacteria may support the AMF for phosphorus acquisition^37^.

Microbial interactions have been suggested as a valuable characterization of the stability of the microbial community in the system^38^. In addition, the bacterial-fungal interactions have been suggested as key drivers of many ecosystem functions, and their study may be relevant for plant health and development^39^. In general, the BA source, which presented the largest values for sugarcane total dry matter, showed the smallest number of microbial connections along with the highest Positive/Negative ratio. The decrease in microbial interactions has been only associated with systems under disturbance^26^. For example, contaminated soils showed smaller microbial connections than those of non-contaminated soils^40^. However, it is important to emphasize that the use of different phosphate sources could not be considered an extreme disturbance, such as pollution^41^ or land use change^42^. Thus, we suggest a different dynamic for this system with low disturbance, such as lack of (control and CA source) or excess of fertilization (SS source). The percentage of P_2_O_5_ may increase microbial interactions between bacteria and fungi microorganisms (bacteria-fungi). Considering the best supply of P for plants under BA, and the selective pressure of the source (evidenced in the PCoA), we can suggest that the demand of microbial interactions is increased in this scenario when compared to the others.

Exploring network analysis, the Positive/Negative ratio has been used to indicate the level of competition within the microbial system^43^, where a ratio smaller than 1 indicates a competition^44^. The results indicated that the BA source induced the lowest level of competition (obtained by Positive/Negative ratio), and strongest system stability, as indicated by Ma (2018). Other interesting patterns of microbial networks were observed across the phosphate sources. The number of bacterial-bacterial interactions increased with the percentage of P_2_O_5_ in fertilizers and, in the same direction, while the number of fungi-fungi connections decreased. A possible explanation may be related to the same strategy suggested for the decrease in mycorrhizal colonization of plants under fertilization^45^. The increase of the phosphorus content may promote an acidulation in the system, which may benefit the bacterial community, increasing their interactions, and drawing back the soil fungal community.

We evaluated using exponential regressions the correlation between the bacteria-fungi connections, the phosphate sources, and total dry matter of sugarcane. The regression curves indicated that the largest value of total dry matter might be achieved using a fertilizer with 11.09% of P_2_O_5_. However, caution must be raised here since a diverse number of other biotic and abiotic factors could affect sugarcane performance and dry matter accumulation. Our results also suggest that a large amount of total dry matter is not necessarily obtained from phosphate fertilizer sources of high percentage of P_2_O_5_, and the bacterial-fungal interactions may help to identify better fertilizer sources and assess an optimal level.

Despite these promising indications, the patterns identified for bacterial-bacterial and bacterial-fungal interactions across phosphate sources remain unclear. Thus, studies implementing the microbial interaction analyses to indicate system stability are recommended. This study was designed to determine the effect of different phosphate sources on the structure of soil bacterial and fungal communities. The findings raised critical theoretical issues that will affect the strategies used for crop fertilization and emphasized the need to advance our knowledge regarding the use of soil microbial community in managing adequately agricultural systems.

## Methods

### Experimental design

The study was performed in a greenhouse at the Soil Science Department of the Luiz de Queiroz College of Agriculture, University of São Paulo, Piracicaba – SP, from April to July 2016. The average temperature during this period ranged from 20 to 27 °C. The soil used in this study had 9.0 mg dm^−3^ of phosphorus, which is considered low for sugarcane cultivation^46^. The chemical composition of the soil used in the experiment is introduced in the Supplementary Table S1. The phosphorus sources were selected based on their percentage of P_2_O_5_, which included Simple Superphosphate (SS, 18.0% of P_2_O_5_), Catalão rock phosphate – Brazil (CA, 2.93% of P_2_O_5_), and Bayóvar reactive rock phosphate - Peru (BA, 14.0% of P_2_O_5_), as well as a control treatment with no addition of phosphorus. All the treatments were tested with and without AMF inoculation. The inoculum was made of spores of *Rhizophagus clarus* isolates supplied by the Laboratory of Soil Microbiology at the University (ESALQ/USP).

Eight treatments (three phosphorus sources plus one control, with/without AMF inoculation) were applied to 3-kg pots containing sugarcane plants. Each one of the eight pots constituted an experimental unit, from which one soil sample was collected for the further analysis. We used four replicates to represent each treatment, and the ensuing 32 pots were randomly distributed in the greenhouse, reducing possible variations such as internal variations in temperature or moisture. The sugarcane plants (cultivar CTC2) were pre-germinated in sterile vermiculite for 20 days, and then transplanted in the pots (one sugarcane plant per pot). The inoculation with mycorrhizal fungi was made one week after transplanting, adding the equivalent of 800 spores per pot, diluted in 10 mL of water^47^. The pots were filled with 3 kg of dry and sieved (2 mm) soil. For sugarcane, the recommendation level for phosphorus is 60 mg of P_2_O_5_ per kg of soil^46^. To achieve the recommendation level for the phosphorus, 1 g of simple superphosphate (SS), 1.28 g of Bayóvar reactive rock phosphate (BA), and 6.14 g of Catalão rock phosphate (CA) were applied. During the experiment, the pots were daily irrigated with distilled water, maintaining the soil moisture at the average of 80% (g/g).

### Plant and soil analysis

The sampling of the root and shoot systems, as well as the rhizosphere soil, was performed at 120 days after transplanting. The accumulation of nutrients N, P and K in the shoot was estimated by the total dry matter accumulated and nutrient content in tissues^48^. The total dry matter of sugarcane plants was obtained by the sum of root and shoot systems after oven drying at 65 °C until constant weight. The rhizosphere soil was considered as the remaining soil after the brief stirring of the plant root system, which was sampled after an intensive agitation and used to determine the diversity of the microbial community. The root system was washed and subsequently sampled, removing fragments of about 1 cm length of thin roots, representing the whole root system colonized by the mycorrhizal fungi. The root sampled was clarified with KOH (10%) and stained with trypan blue in lactoglycerol 0.05%^49^. The percentage of root system colonized by the mycorrhizal fungi was obtained by the method described by Giovannetti and Mosse^50^, which is based on the presence/absence of AMF infection, and percentage of cortex colonized by the AMF.

### DNA extraction and DGGE analysis of bacteria and fungi

DNA extraction of soil samples was performed with the commercial kit PowerSoil DNA Isolation (MoBio, Carlsbad, USA) according to the manufacturer’s instructions. Denaturing Gradient Gel Electrophoresis (DGGE) was performed to verify the structure of bacterial and fungal communities. DNA fingerprinting analysis, such as DGGE, showed relevance in the past^51^, and still have been used in different microbial studies, such as soil microbial communities associated with metal-tolerant plants^52^, or soil fungal community structure in the rhizosphere and bulk soil^53^. DGGE analysis allows for the monitoring of microbial communities under multiple environmental parameters^54^. Despite other advance molecular methods, DGGE analysis is an important tool to evaluate the effect of different phosphorus source on soil bacterial and fungal community profiles. The bacterial community was evaluated by primers 24f (5′ -GAGAGTTTGATCCTGGCTCA -3′) and 1492r (5′-TACGGYTACCTTG TTACGACT-3′), followed by primers 1386f and 968r (with GC-clamp), both reaction was performed using the conditions described by Heuer *et al*.^55^. To determine the structure of the fungal community, we amplified the intergenic region 1 (ITS1) with primers sets EF4 (5′-GAAAGGGRTGTATTT ATTTAG-3′) and ITS4 (5′-TCCTCCGCTTATTGATATGC -3′), followed by primers ITS1f GC (5′-CTTGGTCATTTAGAGGAAGTAA-3′) and ITS2 (5′-GCTGCGTTCTTCATCGATGC-3′), according to the conditions described by Anderson *et al*.^56^. The PCR products were loaded on polyacrylamide gels (8% for fungi and 6% for bacteria) with denaturing gradient of 15–65% for the fungi and 40–60% for the bacterial (100% denaturant according to 7 M urea and 40% formamide in 1x TAE buffer) and then were run for 16 h at 90 V and 60 °C in 1x TAE Buffer. After electrophoresis, 50 ml of the solution SYBR GREEN^TM^ (Invitrogen, UK) 600x concentrate in DMSO was applied on the gels, revealing the microbial profiles for each sample. The gel was visualized and analysed using ImageQuant TL 7.0 (GE Healthcare Life Sciences).

### Statistical analysis

The statistical analyses were performed using the R software^57^ with the packages ‘*vegan*’, ‘*igraph*’, and ‘*plot3D*’. Total dry matter and percentage of root colonization were tested for normality and subjected to analysis of variance (ANOVA), and their means were compared using the Tukey test (*p* < *0*.*05*). The structure of bacterial and fungal communities obtained from DGGE analysis was evaluated using principal component analysis (PCoA) across phosphate sources in the presence or absence of inoculation. The distance between the microbial matrices was performed using “Bray-Curtis” similarity. PERMANOVA analyses evaluated the correlation between the structure of bacterial and fungal communities and phosphate sources, using the formula,

1 $$dist\,(microbial\,table)\sim phosphate\,sources+Inoculum\,addition$$

The correlations were performed using ‘Bray-Curtis’ as the microbial distance (dist), and 9999 permutations, for bacterial and fungal communities. The profiles obtained by DDGE analysis were used to evaluate the potential microbial interactions for each phosphate source. The microbial groups were filtered by minimal of 25% frequency across the samples, which eliminated singleton groups across the microbial profiles. The microbial connections of each bacterial and fungal group (band obtained in DGGE gel) was evaluated using Spearman and Pearson correlation (*reviewed by* Faust and Raes)^58^, considering p-value < 0.05 for both correlations. The topological properties of each network analysis, such as the number of connections, and Positive/Negative ratio^43^, were performed using the R package *‘igraph’*^59^. The exponential curve correlations, including the R^2^, p-value and residual standard error (RSE) were performed using the R package ‘*stats*’. The best fits for linear regression were based on the RSE, as suggested by Itakura^60^.

## Supplementary information

Supplementary Material
