# Supplementary material for: Phosphorus source driving the soil microbial interactions and improving sugarcane development
Source: Sci Rep. 2019 Mar 13;9:4400. doi: 10.1038/s41598-019-40910-1 (PMC6416284; doi:10.1038/s41598-019-40910-1)
Supplement: Supplementary file 1 — Supplementary Material [file 41598_2019_40910_MOESM1_ESM.docx]

***Supplementary Material***

***Title: Phosphorus source driving the soil microbial interactions and improving sugarcane development***

***Authors:*** *Thiago Gumiere, Alain N. Rousseau, Diogo Paes da Costa, Alice Cassetari, Simone Raposo Cotta, Fernando Dini Andreote, Silvio J. Gumiere, Paulo Sergio Pavinato*

*
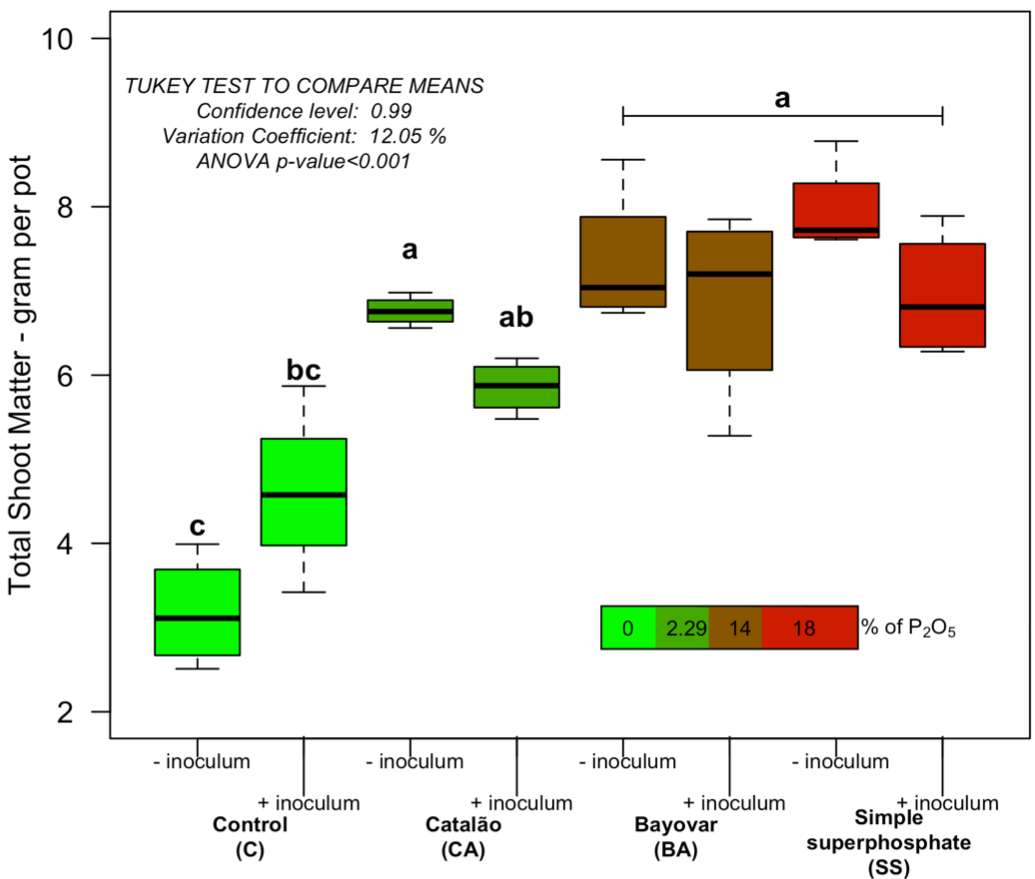
*

Supplementary Figure S1. Boxplot of total shoot dry matter (grams per pot) of sugarcane plants across phosphate sources in the presence and absence of mycorrhizal fungi inoculation. The bar color indicates the % of P_2_O_5_ per each phosphorus source. The averages were compared by Tukey test (p-value<0.001).


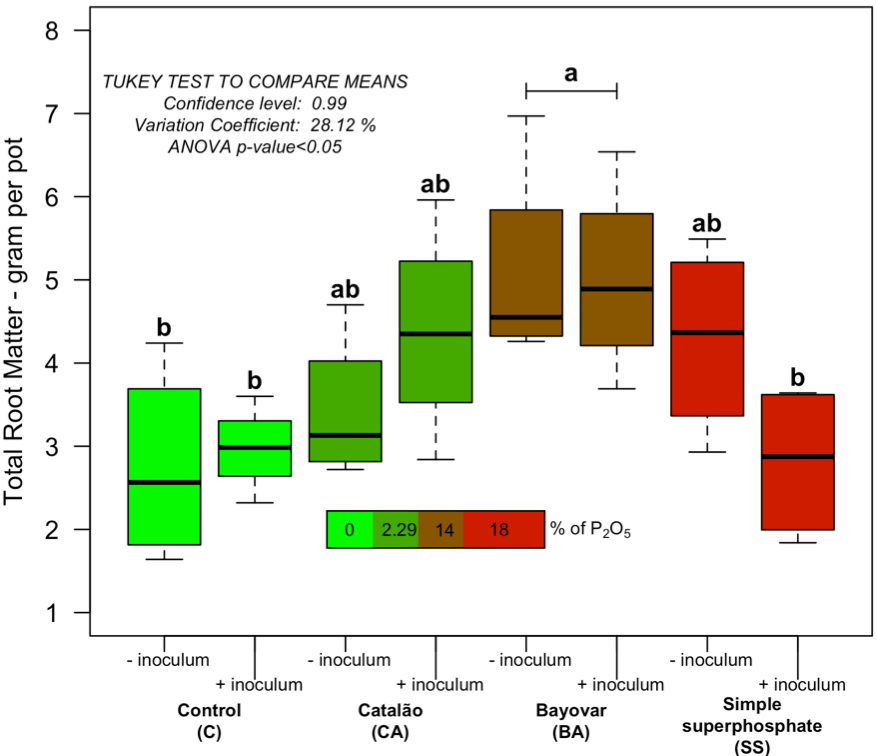


Supplementary Figure S2. Boxplot of total root dry matter (grams per pot) of sugarcane plants across phosphate sources in the presence and absence of mycorrhizal fungi inoculation. The bar color indicates the % of P_2_O_5_ soluble per each phosphorus source. The averages were compared by Tukey test (p-value<0.05).


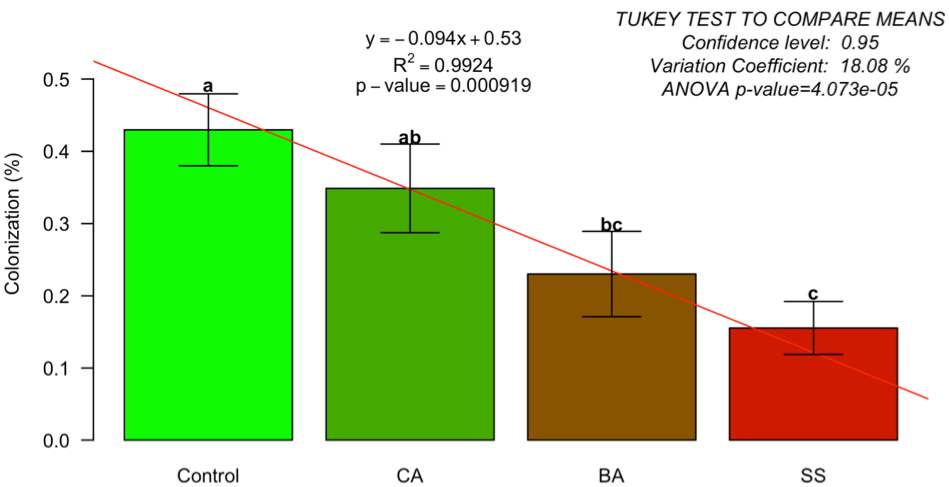


Supplementary Figure S3. Percentage of sugarcane roots colonized by arbuscular mycorrhizal fungi of each phosphate source used: Control; CA – Catalão rock phosphate; BA – Bayovar rock phosphate; SS – simple superphosphate. The averages were compared by Tukey test considering p-value <0.05. The decreased pattern is indicated by the linear regression line (red line).


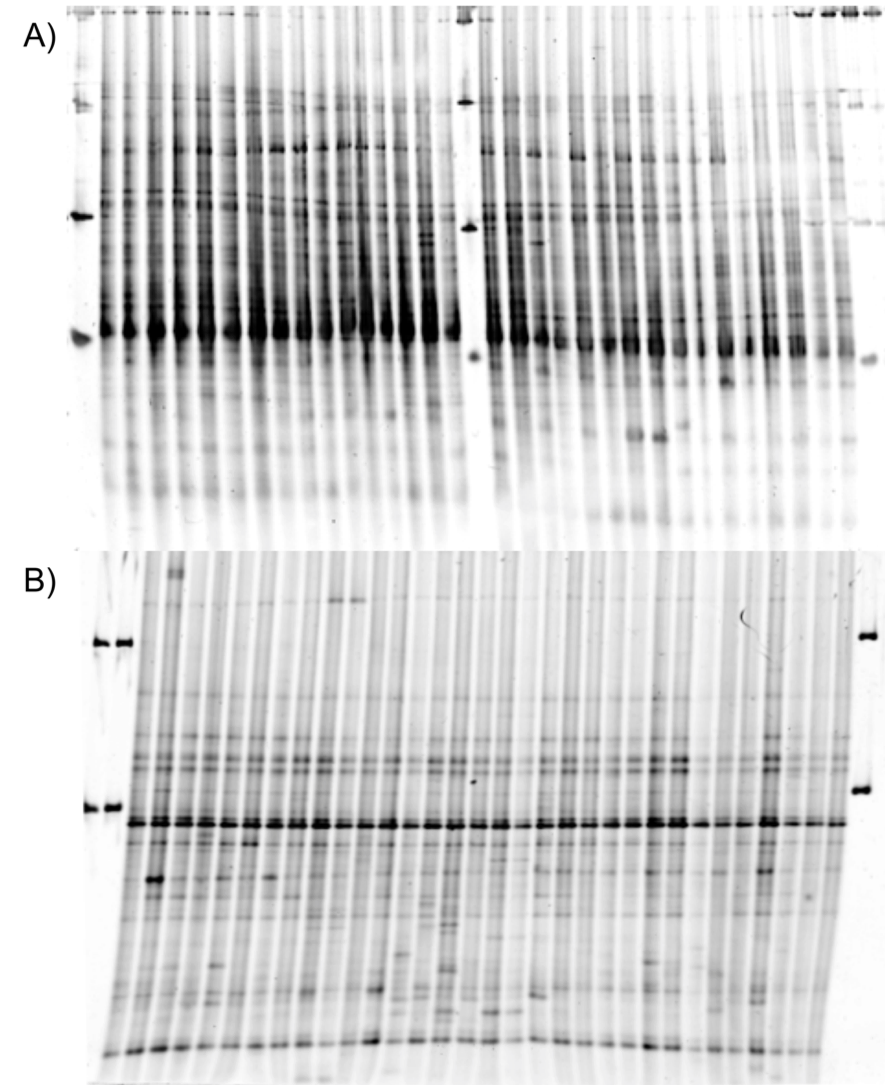


Supplementary Figure S4. DGGE acrylamide gel of bacterial (A) and fungal (B) community.


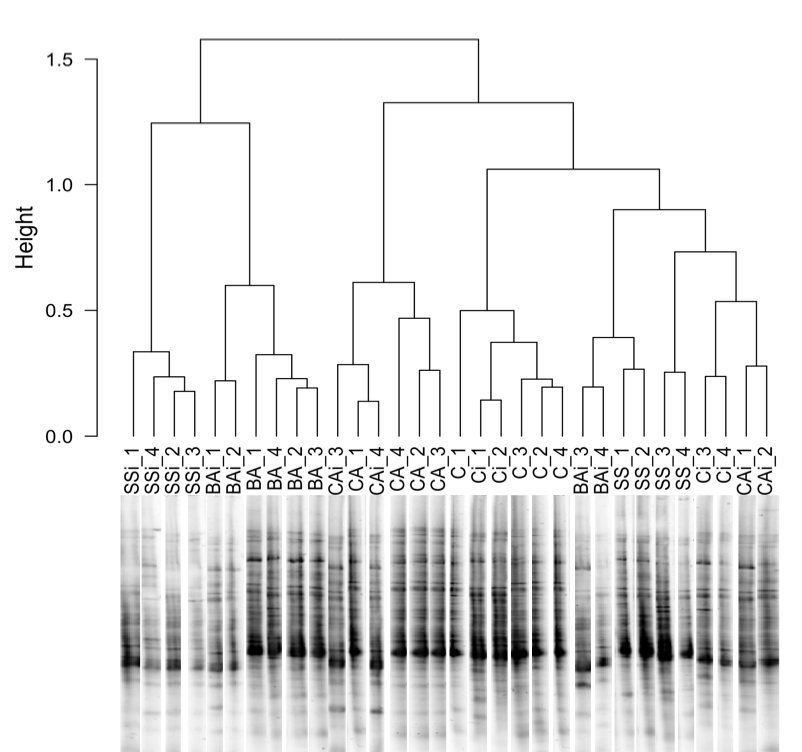


Supplementary Figure S5. Cluster analyses and DGGE acrylamide gel of bacterial community. The phosphate sources are identified as C – Control; CA – Catalão rock phosphate; BA – Bayovar rock phosphate; SS – simple superphosphate. The treatments with arbuscular mycorrhizal inoculation were identified as “_i”, e.g. C_i – Control with inoculum.


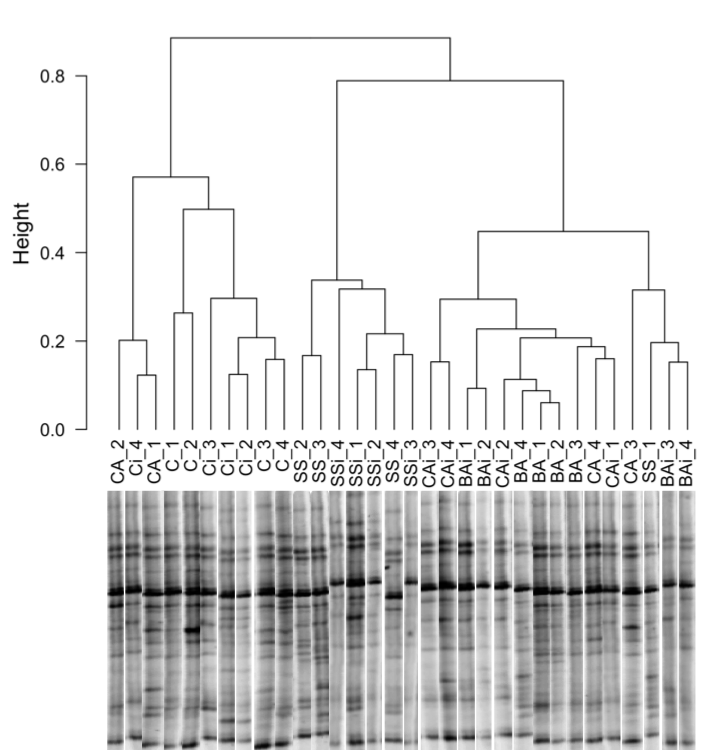


Supplementary Figure S6. Cluster analyses and DGGE acrylamide gel of fungal community. The phosphate sources are identified as C – Control; CA – Catalão rock phosphate; BA – Bayovar rock phosphate; SS – simple superphosphate. The treatments with arbuscular mycorrhizal inoculation were identified as “_i”, e.g. C_i – Control with inoculum.


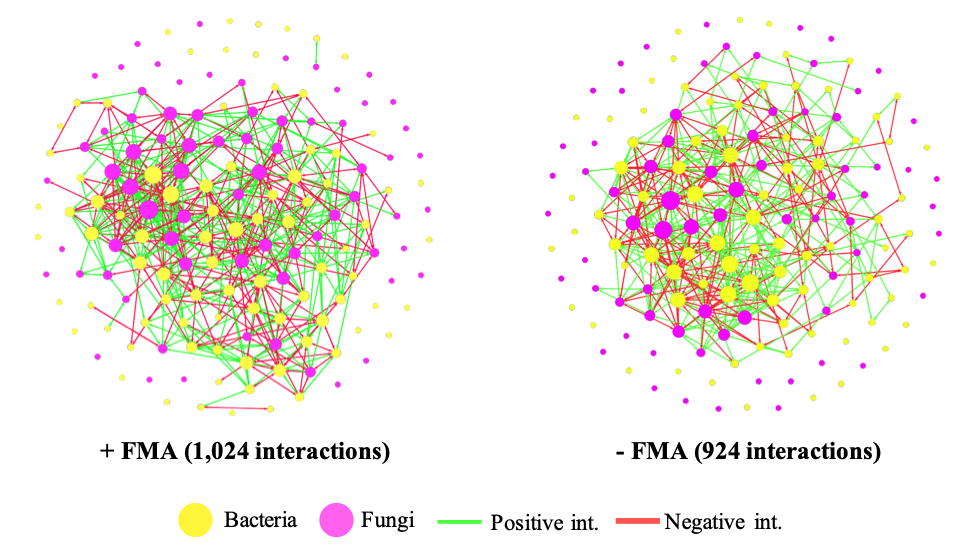


Supplementary Figure S7 – The network of bacterial and fungal interaction obtained from Pearson and Spearman correlations (p-value<0.05) of samples in the presence and absence of inoculation. The positive correlations are indicated by green lines and negative correlation by red lines. The total number of microbial interactions is indicated by the presence and absence of FMA.


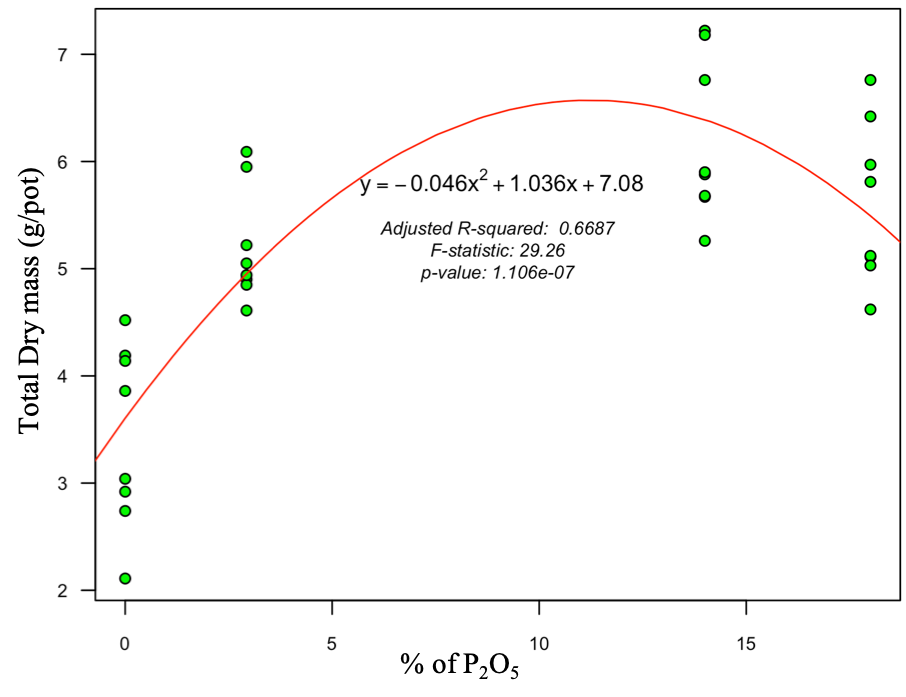


Supplementary Figure S8 – Exponential regression curve correlating the percentage of P_2_O_5_ in phosphate sources and sugarcane total dry matter. The green points represent the observed data, and trend line is indicated in red (p-value<0.05).


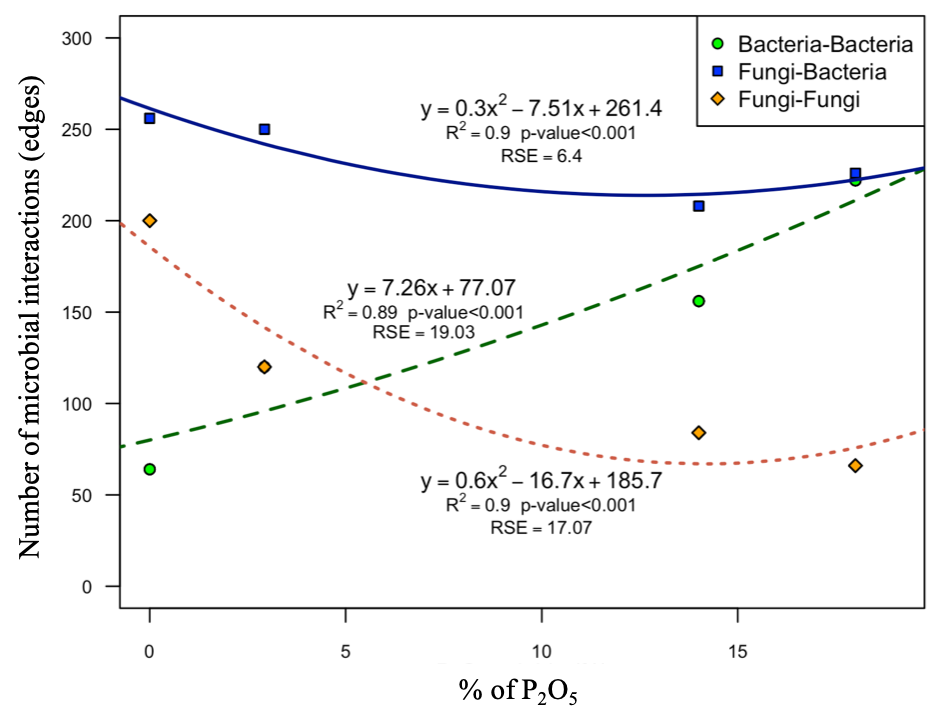
 Supplementary Figure S9. Regression exponential curve correlating the percentage of P_2_O_5_ in phosphate sources and microbial interactions of Bacteria-Bacteria (green points), Bacteria-Fungi (blue points) and Fungi-Fungi (orange points). Trendlines were included for each microbial interaction group, such as bacteria-bacteria, fungi-fungi, and bacteria-fungi

Supplementary Table S1 – Initial chemical parameters of the soil used in the experiment.

| **Chemical parameters** | **Values** |
| --- | --- |
| *pH (CaCl_2_)* | 5 |
| *O.M (g.dm^-3^)* | 18 |
| *P-resin (mg.dm^-3^)* | 9 |
| *K (mmol_c_.dm^-3^)* | 1.8 |
| *Ca (mmol_c_.dm^-3^)* | 7 |
| *Mg (mmol_c_.dm^-3^)* | 5 |
| *H+Al (mmol_c_.dm^-3^)* | 20 |
| *Al (mmol_c_.dm^-3^)* | 0 |
| *Base Sum (mmol_c_.dm^-3^)* | 14 |
| *CEC(mmol_c_.dm^-3^)* | 34 |
| *Sat. Base (V%)* | 41 |
| *Sat. Al (m%)* | 0 |
| *S-SO_4_ (mg.dm^-3^)* | 8 |

Supplementary Table S2 – Phosphorus, nitrogen and potassium content (g kg^-1^) in sugarcane leaves under phosphate sources and in the presence and absence of arbuscular mycorrhizal inoculation.

|  | **Element contents (g kg^-1^ of dry leaf)** | | |
| --- | --- | --- | --- |
| **Treatments** | **Phosphorus** | **Nitrogen** | **Potassium** |
| Control | 0.69 *c** | 10.71 *a* | 12.43 *b* |
| CA | 1.14 *b* | 9.14 *b* | 14.15 *a* |
| BA | 1.27 *ab* | 8.94 *b* | 12.43 *b* |
| SS | 1.31 *a* | 7.97 *b* | 13.20 a*b* |
|  |  |  |  |
| + inoculum | 1.11 *a* | 9.31 *a* | 13.48 *a* |
| - inoculum | 1.10 *a* | 9.07 *a* | 12.62 *b* |

*The column averages were compared by Tukey test (p-value <0.05) separated for phosphate sources and for arbuscular mycorrhizal inoculation.

Supplementary Table S3 – Correlation between the phosphorus source (control, CA, BA, and SS) and FMA inoculum (+ and -) with bacterial and fungal communities, using PERMANOVA analysis, “Bray-Curtis” similarity, and 9,999 permutations.

|  | | | | | | |  |
| --- | --- | --- | --- | --- | --- | --- | --- |
| **Bacterial community** | **Df** | **Sums Of Sqs.** | **Mean Sqs** | **F. Model** | **R^2^** | **Pr(>F)** | |
| **P_2_O_5_ sources** | 3 | 1.39 | 0.46 | 7.84 | 39.1% | 0.001^***^ | |
| **Inoculum** | 16 | 1.46 | 0.09 | 1.55 | 41.0% | 0.003^**^ | |
| Residuals | 12 | 0.71 | 0.06 |  | 19.9% |  | |
| Total | 31 | 3.55 |  |  | 100% |  | |
|  | | | | | | |  |
|  | | | | | | |  |
| **Fungal community** | **Df** | **Sums Of Sqs.** | **Mean Sqs** | **F. Model** | **R^2^** | **Pr(>F)** | |
| **P_2_O_5_ sources** | 3 | 0.75 | 0.25 | 8.11 | 45.77% | 0.001^***^ | |
| **Inoculum** | 16 | 0.52 | 0.03 | 1.05 | 31.67% | 0.381^NS^ | |
| Residuals | 12 | 0.37 | 0.03 |  | 22.56% |  | |
| Total | 31 | 1.64 |  |  | 100% |  | |

*Signif. codes: 0.001 ‘***’; 0.01 ‘**’; 0.05 ‘*’; >0.05 ^‘NS’^*
